# Effect of Process Parameters on the Initial Burst Release of Protein-Loaded Alginate Nanospheres

**DOI:** 10.3390/jfb10030042

**Published:** 2019-09-16

**Authors:** Farhana Yasmin, Xiongbiao Chen, B. Frank Eames

**Affiliations:** 1 Division of Biomedical Engineering, College of Engineering, University of Saskatchewan, Saskatoon, SK S7N 5A9, Canada; farhanayasmin133@gmail.com (F.Y.); xbc719@mail.usask.ca (X.C.); 2 Department of Mechanical Engineering, College of Engineering, University of Saskatchewan, Saskatoon, SK S7N 5A9, Canada; 3 Department of Anatomy, Physiology and Pharmacology, College of Medicine, University of Saskatchewan, Saskatoon, SK S7N5E5, Canada

**Keywords:** alginate micro/nanospheres, controlled delivery, protein, initial burst release, protein delivery device, alginate concentration, cross-linking time, drying time, size of nanospheres

## Abstract

The controlled release or delivery of proteins encapsulated in micro/nanospheres is an emerging strategy in regenerative medicine. For this, micro/nanospheres made from alginate have drawn considerable attention for the use as a protein delivery device because of their mild fabrication process, inert nature, non-toxicity and biocompatibility. Though promising, one key issue associated with using alginate micro/nanospheres is the burst release of encapsulated protein at the beginning of the release, which may be responsible for exerting toxic side effects and poor efficiency of the delivery device. To address this issue, this study aimed to investigate the effect of process parameters of fabricating protein-loaded alginate nanospheres on the initial burst release. The alginate nanospheres were prepared via a combination of water-in-oil emulsification and the external gelation method and loaded with bovine serum albumin (BSA) as a model protein. The examined process parameters included alginate concentration, ionic cross-linking time and drying time. Once fabricated, the nanospheres were then subjected to the examination of BSA release, as well as the characterization of their morphology, size, and encapsulation efficiency. Our results revealed that by properly adjusting the process parameters, the initial burst release can be reduced by 13%. Taken together, our study demonstrates that regulating process parameters of fabricating alginate nanospheres is a possible means to reduce the initial burst release.

## 1. Introduction

The delivery of proteins holds a great promise in regenerative medicine because of the ability of proteins to improve tissue regeneration and cure several diseases [1,2]. However, one of the major challenges yet to overcome is to spatiotemporally control the release rate of proteins [3]. Currently, micro/nanospheres have shown great promise as a protein or drug delivery device in regenerative medicine [2,4,5,6]. The use of micro/nanospheres protects the proteins or other encapsulating molecules from denaturation and also enables the release rate to be tailored for the need of different applications [7,8]. Most importantly, using micro/nanospheres allows the spatial and temporal control over the release of proteins or other encapsulating molecules at the target site and for a long period of time, thereby, increasing the effectiveness [9,10].

Among various polymer-based micro/nanospheres, alginate micro/nanospheres have been widely used as a protein delivery device in some studies [11,12,13,14,15]. The reason of increasing interest in using alginate as a delivery device is most likely due to its excellent biocompatibility and easy processing condition, which allows for the incorporation of proteins or other molecules without denaturation [12,16,17]. However, the problems associated with using alginates as a protein delivery device include the alginates’ inherent high porosity and instability at a high pH medium, resulting in the burst release of encapsulated proteins or other molecules at the beginning of the release [14,15,18]. In most cases, this burst release is undesired due to the toxic side effects and decreased efficiency of the delivery device [3,18,19]. The burst release occurs due to surface protein desorption and diffusion from the outer surface of micro/nanospheres and is proportional to the surface area-to-volume ratio exposed to the release medium [20,21]. This is particularly true for the porous micro/nanospheres given that they have a larger surface area-to-volume ratio and consequently high permeability to release the medium, resulting in an increased initial burst release [22]. 

In order to design an alginate-based protein delivery device and reduce the burst release, it is important to understand the chemistry of alginate. Alginate is a naturally occurring anionic copolymer composed of 1–4 glycosidically linked mannuronic acid (M-unit) and guluronic acid (G-unit) [23]. The function of alginate as a protein delivery device depends on the molecular weight (MW), and the relative percentage of the G and M units of alginate [23]. Only the G-units of an alginate participate during ionic cross-linking with Ca^2+^ and with the increase of G-units, the degree of cross-linking increases [23]. Therefore, compared to the low MW alginates, the high MW alginates have a relatively higher percentage of G-units and are more effective in retarding protein diffusion [18]. However, the alginate solution prepared from high MW polymer is viscous, which might cause damage to proteins from the high shear force induced during mixing [23]. Therefore, the MW of alginate should be carefully selected during designing an alginate-based protein delivery device.

There are many other ways that can be used to prolong the release rate and to reduce the initial burst release. Among them, coating or reinforcing with other polymers have been often used [24,25]. However, coating or reinforcing adds extra processing steps/cost and also might result in protein loss due to an incomplete protein release [24,26]. As such, alternative approaches to reduce the initial burst release are of interest. Earlier studies suggested that the polymer concentration [21,27,28], crosslinking time [29,30,31] and drying time [32] are of importance to affect the release rate of encapsulated molecules. In these studies, however, most often the encapsulating molecules were not proteins but small MW drugs, dye and other chemicals [28,30,32,33,34,35]. To the best of the authors’ knowledge, the effect of the alginate concentration, cross-linking time or drying time on the initial burst release of the protein has been missing in the literature. Evaluating the effect of these process parameters is important to better understand which factors are playing dominant roles to control the initial burst release. To fill the gap, this study aimed to develop the alginate nanospheres as a protein delivery device, with a focus on identifying the effect of the nanospheres’ process parameters on the initial burst release. For this, this study hypothesized that, increasing the alginate concentration, cross-linking time or drying time allows for reduction of the initial burst release. In this study, bovine serum albumin (BSA) was used as a model protein in this study.

## 2. Materials and Methods

### 2.1. Materials

The materials were low viscosity Sodium Alginate (Cat #B25266, lot #R24D027) from Alfa Aesar by Thermo Fisher scientific (Heysham, UK), Paraffin oil, Span 80, BSA (A2153), Dulbecco’s modified Eagle’s medium (D5648) and Penicillin-Streptomycin from Sigma Aldrich (Oakville, Canada), 24 and 96 well plates from Thermo Fisher Scientific, isopropyl alcohol, double distilled water (DDW), calcium chloride (CaCl_2_), Phosphate buffered saline (PBS) and a Bradford assay reagent.

### 2.2. Preparation of Protein Loaded Alginate Nanospheres

**Figure 1:**
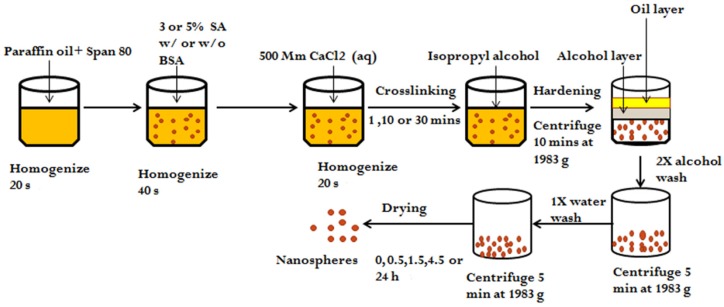
Schematic diagram of nanosphere fabrication.

In order to prepare the protein loaded alginate nanospheres, a combination of the without emulsification and external gelation method was used as reported in [36]. Briefly, sodium alginate (SA) was dissolved in DDW with the help of a magnetic stirrer to get an alginate concentration of either 3% or 5%. BSA, a model protein was then added into the alginate solution and mixed completely with the help of a magnetic stirrer to get a protein concentration of 300 µg/mL of alginate. The initial loading ratio of protein and alginate were 1:100 and 1:167 for 3 and 5% alginate respectively. Then, in a 24 well plate, 50 µL of a nonionic Span 80 surfactant was added in 1 mL of paraffin oil to get a Span 80 concentration of 5%. The mixture was homogenized at 5000 rpm for 20 s using a probe type tissue homogenizer (Fisherbrand™ 850 homogenizer (Fisher Scientific, Singapore) with powerful 850-watt motor, probe dimension: 7 × 110 mm). Next, 300 µL of aqueous alginate/BSA solution was added into the non-aqueous paraffin oil/Span 80 mixture and was homogenized again for another 40 s to break down the solution into small emulsion droplets, thereby, forming a water-in-oil emulsion. To cross-link the alginate droplets by ionic gelation, 150 µL of 500 mM CaCl_2_ solution was added externally and the mixture was further homogenized for 20 s. The mixture was then left for cross-linking for 1, 10 or 30 min to form the cross-linked alginate nanospheres. After cross-linking for a specific period of time, 300 µL of pure isopropyl alcohol was added to harden the alginate nanospheres. The resultant nanospheres were then collected by centrifugation at 1983× *g* for 10 min. To remove some of the trapped oil droplets, unreacted reagents, and uncomplexed polymers, the nanospheres were then washed two times with 1 mL isopropyl alcohol and one time with 1 mL DDW. For washing, the nanospheres were resuspended in washing buffer (isopropyl alcohol/ DDW) and then collected by centrifuging at 1983× *g* for 5 min. After fabrication, the nanospheres were either used directly without drying or dried at 37 °C for 0.5, 1.5, 4.5 or 24 h and then used for the nanospheres’ characterization. Alginate interferes during the Bradford assay (see Supplemental Figure S1). Therefore, alginate nanospheres without any BSA were prepared the same way as mentioned above except no BSA was mixed with the alginate and were used as a negative control. The schematic diagram of the fabrication method used to prepare alginate nanospheres is given in Figure 1. It is important to note that three samples from each of two different batches were used during the determination of the effect of each process parameter.

### 2.3. Experimental Design

The process parameters used in the fabrication of BSA-loaded alginate nanospheres is summarized in Table 1, wherein one of the parameters were altered while the others were kept constant. Specifically, the first step of the experiment was to determine the effect of alginate concentration on the initial burst release. Then, the concentration that showed a minimal initial burst release was used in the second step to determine the effect of the cross-linking time on the initial burst release; and eventually the cross-linking time that showed a minimal initial burst release was used, along with the alginate concentration selected in the first step, to determine the effect of drying time on the initial burst release.

### 2.4. Characterization of Alginate Nanospheres

#### 2.4.1. Morphology and Size of Nanospheres

The morphology and the size of alginate nanospheres were examined by using an optical microscope (Zeiss Axioskop 20, Jena, Germany) equipped with a camera. Briefly, the nanospheres were suspended in 1 mL Dulbecco’s Modified Eagle’s Medium (DMEM) (with a value of Ca^2+^ 1.8 mM and pH 7.4). From the suspended nanospheres, 3 µL was taken in a microscope slide and micrographs were taken using the optical microscope. The volume of the suspended nanospheres was kept constant at 3 µL, and 63× magnification was used to take micrographs. The size and distribution pattern of nanospheres, prepared using various processing conditions, were then analyzed from these micrographs using the ImageJ software. The ImageJ software (I.51s, National Institues of Health, Bethesda, MD, USA) provides the area of nanospheres. To determine the size of nanospheres, the area of nanospheres was then used to calculate the diameter of nanospheres using Excel. The distribution pattern of nanospheres prepared using different experimental conditions was analyzed using the StatPlus software (6.1.50, AnalystSoft Inc., Walnut, CA, USA). To produce a histogram, the lower bin limit was set to 100 and the upper bin limit to 2000 with a bin interval of 100 for different experimental conditions. It is important to note that at the very beginning of this study to confirm that the micrograph contains only nanospheres but not any paraffin droplets (used during nanospheres fabrication), a negative control was prepared following the same protocol used for nanospheres fabrication, except no alginate solution was added. Then, the resultant solution was washed 2× with isopropyl alcohol and 1× with water. Afterwards, DMEM was added and micrographs were taken using the optical microscope to ascertain if there were any paraffin oil droplets present in the micrograph. However, no paraffin oil droplets were found under the optical microscope confirming that the spheres present in the micrographs are nanospheres and not any paraffin droplets. Furthermore, the presence of BSA showed no significant effect on the size of alginate nanospheres (see Supplemental Figure S2).

#### 2.4.2. Protein Release from the BSA Loaded Nanospheres In Vitro

In this study, the supernatant protocol [4] (i.e., removing whole supernatant from the same batch of nanospheres at different timepoints) was used to examine the protein release kinetics. Nanospheres were suspended in 1 mL of DMEM (with the same value of Ca^2+^ and pH of DMEM are 1.8 mM, 7.4 respectively, as the ones noted above) containing 1% Penicillin-Streptomycin (PS) and were incubated in a shaking incubator operating at 37 °C and 100 rpm. PS was used in DMEM to prevent the contamination by bacteria or fungi. Four different timepoints, such as 5-h, 24-h, 48-h and 96-h, were selected in order to determine the protein release kinetics. The value of protein release at the 0-h timepoint, i.e., immediately after nanosphere fabrication was considered as 0, while the protein release at the other predetermined timepoints were characterized from 1 mL supernatant collected by means of centrifugation at 1983 × g for 10 min and 16,873× *g* for 15 min. Specifically, nanospheres were initially collected at a low centrifugation speed so as to prevent nanospheres from damage that may occur at high speeds. However, due to the use of such a low centrifugation speed, some of the nanospheres (approximately 10%) were still present in the collected supernatant. To collect the remaining nanospheres from the supernatant, the supernatant was further centrifuged at a high centrifugation speed (16,873× *g*) for 15 min. The collected nanospheres were then re-suspended in 1 mL fresh DMEM and placed into a shaking incubator. To get a clear supernatant solution, the collected supernatant was centrifuged again at 16,873× *g* for 15 min and used for analysis. The process was repeated until the end of the experiment. The protein content in the collected supernatant was analyzed using the Bradford protein assay which has an absorption maximum at 595 nm. The linear concentration range used was 1.56–100 µg/mL of protein, using BSA as the standard protein molecule. To normalize the protein release kinetics, the protein released at each timepoints was divided by the nanospheres’ weight to obtain the protein release per unit of nanospheres. The protein release at different timepoints was then calculated as a percentage of the total protein release.

#### 2.4.3. Determination of Initial Burst Release

To characterize the initial burst release, the BSA released at the 5-h timepoint was used. To determine the initial burst release, the total released protein at the 96-h timepoint was considered as 100% release. The percentage of the total released protein at the 5-h timepoint was calculated using the following equation:

(1) $$Burst\;Release\;\left(\%\right)=\frac{Percentage\;of\;protein\;released\;\mathrm{per}\;\mathrm{mg}\;\mathrm{of}\;\mathrm{nanospheres}\;at\;5\;hour\;timepoint}{Percentage\;of\;total\;released\;protein\;per\;mg\;of\;nanospheres\;\;after\;96\;hour}\times 100$$

#### 2.4.4. Determination of Encapsulation Efficiency or Total Release

The encapsulation efficiency (EE%) or the total release percentage of the nanospheres was calculated by determining the total amount of BSA released after 96 h. The EE% or total release percentage was determined using the following equation:

(2) $$Encapsulation\;Efficiency/\;Total\;Release\;\left(\%\right)=\;\frac{Mass\;of\;protein\;released\;from\;total\;amount\;of\;recovered\;nanosheres\;after\;96\;hours}{Mass\;of\;protein\;added\;in\;each\;formulation}\times 100,$$

In the present study, since no BSA release was observed after 96 h, the total amount of BSA released at 96-h timepoint was considered as the total amount of the encapsulated protein, as no BSA release after 96 h was found (see Supplemental Figure S3). It is recalled that BSA was added in the alginate solution at a concentration of 300 µg/mL of alginate and 300 µL of the protein containing alginate solution was used to prepare each sample. Thus, the total amount of BSA initially added to each formulation was considered as 90 µg. To evaluate the EE% or the percentage of total protein release, the nanospheres were suspended in DMEM containing PS and then incubated in the shaking incubator (operating at 100 rpm speed and 37 °C) for 96 h. 

### 2.5. Statistical Analysis

In this study, the statistical analysis was performed using SPSS V.22 (SPSS, Chicago, IL, USA). The distribution of data obtained using various processing conditions were tested for normality using the Shapiro-Wilk test. During determination of the effect of each process parameter, if the data fell into normal distribution, the independent-sample t-test was performed when the effect of two independent groups were compared. The one-way analysis of variance (ANOVA) was performed when the effect of more than 2 independent groups were compared. In the case of one-way ANOVA, when significance was found, the Tukey’s multiple comparison test was utilized to assess the difference occurring between the groups. During determination of the effect of each process parameter, if the data did not fall into normal distribution, the Mann-Whitney U test (equivalent to the independent-sample t-test) was performed when the effect of two independent groups were compared and the Kruskal Wallis test (equivalent to one-way ANOVA) was used when more than two independent groups were compared. In the case of Kruskal Wallis test, when significance was shown, the Dunn’s multiple comparison test with the Bonferroni correction was utilized to assess where the difference occurred between the groups. All the data are presented as the mean ± SEM (standard error of the mean). A difference was considered statistically significant for p ≤ 0.05.

## 3. Results

### 3.1. Effect of Alginate Concentration

#### 3.1.1. Morphology and Size of Nanospheres

**Figure 2:**
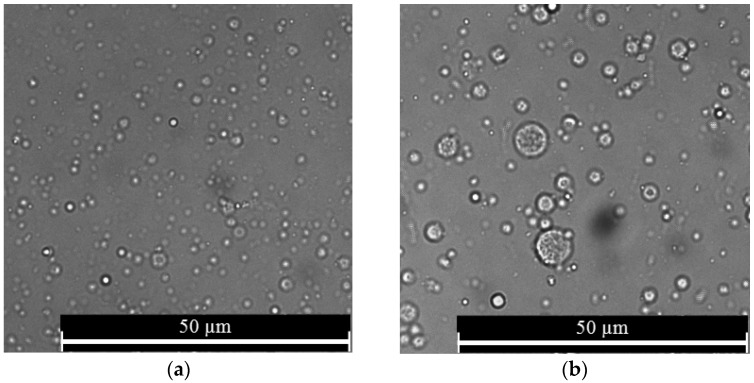
Morphology of alginate nanospheres prepared with (**a**) 3%; and (**b**) 5% alginate. Optical microscope with a 63× magnification was used to take all the micrographs.

**Figure 3:**
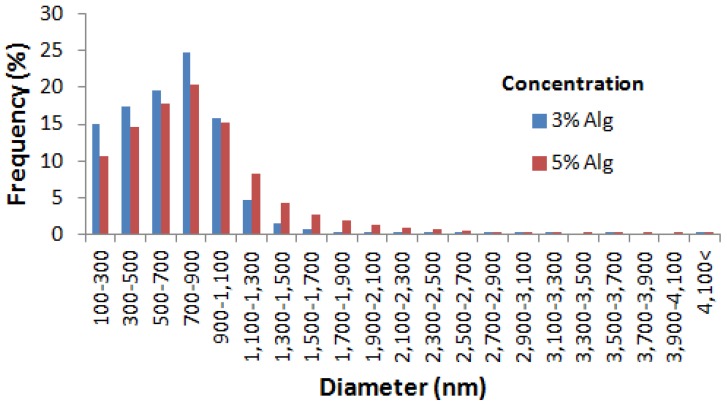
Comparison of the size distribution pattern of alginate nanospheres prepared using different alginate concentrations.

**Figure 4:**
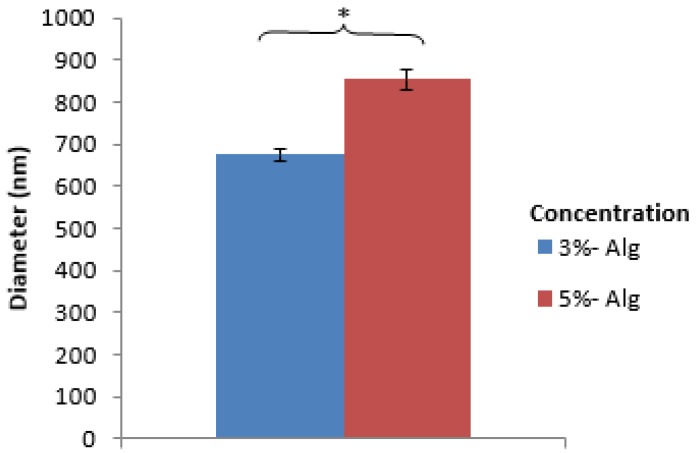
Comparison of the mean diameter of alginate nanospheres prepared using different alginate concentrations (*: Indicates significant difference between groups).

It was observed that nanospheres prepared using various alginate concentrations were spherical in shape (Figure 2). Further, the nanospheres prepared using a higher alginate concentration had a higher apparent contrast compared to their lower alginate concentration counterparts. In addition, both the 3% and 5% alginate resulted in nanospheres having a normal size distribution pattern with most of the nanospheres being in the range from 700 to 900 nm (Figure 3). The analysis of the particle size indicated that the variation of the alginate concentration had a significant effect on the mean diameter of nanospheres (Figure 4).

#### 3.1.2. Protein Release Kinetics and Initial Burst Release

**Figure 5:**
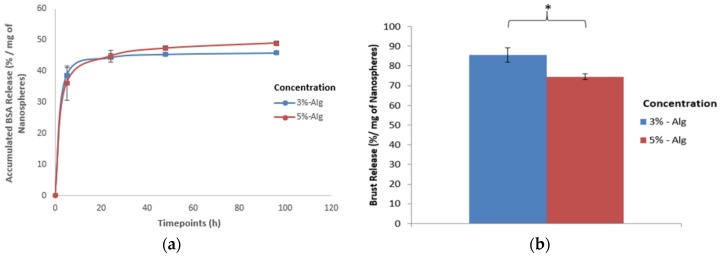
Effect of alginate concentration on the (**a**) protein release kinetics and (**b**) initial burst release of alginate nanospheres (*: Indicates significant difference between groups).

The effect of alginate concentration on the in vitro protein release kinetics and burst release is shown in Figure 5. The alginate concentration changed from 3% to 5% and the cross-linking time and drying time were kept constant at 10 min and 0 h respectively. It was found that the protein release profile (Figure 5a) varied slightly with the change of the alginate concentration. Particularly, with the increase in the alginate concentration, the protein release at the initial timepoint (i.e., 5 h) decreased and the protein released in total (i.e., 96 h) increased. However, the effect was not significant.

When the effect of the alginate concentration on the initial burst release was calculated using Equation (1) (Figure 5b), it was found that increasing the alginate concentration from 3% to 5% showed a significant decrease in the initial burst release (Figure 5b). With the increase of alginate concentration from 3% to 5%, this decreased the initial burst release from 86 ± 3.62 to 74 ± 1.53%.

#### 3.1.3. Encapsulation Efficiency

The effect of alginate concentration on the EE% was also tested in this study, and with the increase of alginate concentration from 3% to 5%, the EE% increased slightly from 47 ± 4.16 to 51 ± 8.09%. However, the increased alginate concentration showed no significant difference in the EE% of the alginate nanospheres.

### 3.2. Effect of Cross-Linking Time

#### 3.2.1. Morphology and Size of Nanospheres

**Figure 6:**
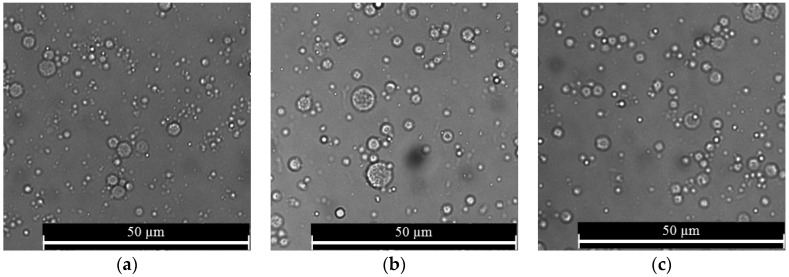
Morphology of alginate nanospheres prepared with (**a**) 1-min cross-linking time; (**b**) 10-min cross-linking time; and (**c**) 30-min cross-linking time. Optical microscope with a 63× magnification was used to take all micrographs.

**Figure 7:**
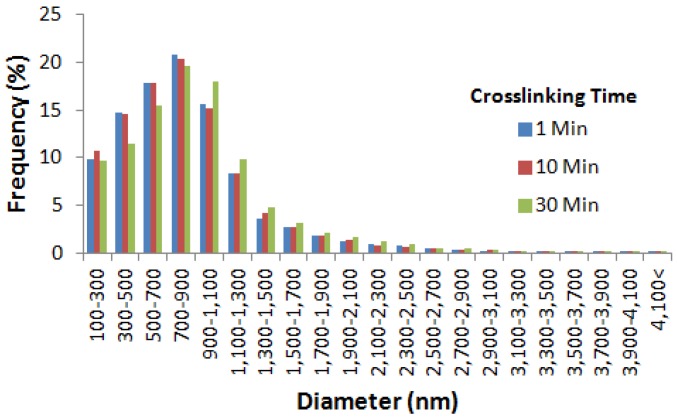
Size distribution pattern of alginate nanospheres prepared using different cross-linking times.

**Figure 8:**
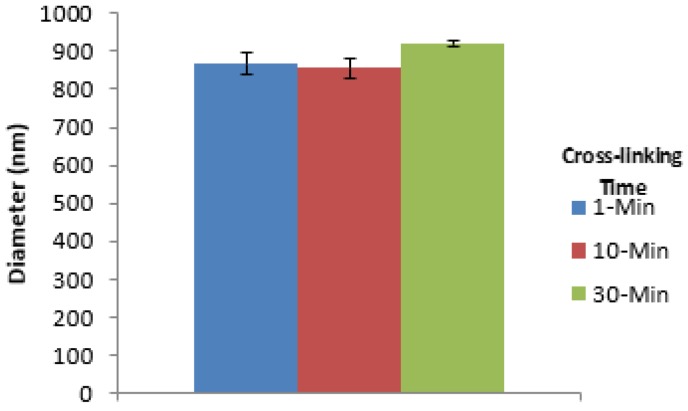
Comparison of the mean diameter of alginate nanospheres prepared using different cross-linking times.

During determination of the effect of ionic cross-linking time on the morphology, size, and distribution pattern of nanospheres, freshly prepared alginate nanospheres prepared using the 5% (w/v) alginate were used without drying (0-h drying time). Nanospheres prepared using various cross-linking times were discrete and spherical in shape with nanospheres aggregation at some of the places (Figure 6). Nanospheres prepared using different cross-linking times showed a normal distribution pattern implying homogeneity (Figure 7). For different cross-linking times, most of the nanospheres were in the range from 700 to 900 nm. The results indicated that with the increase of cross-linking time from 1 to 10 min, the mean diameter of alginate nanospheres decreased very slightly and then increased again when the cross-linking time increased to 30 min (Figure 8). However, the effect of cross-linking time on the mean diameter of nanospheres was not statistically significant.

#### 3.2.2. Protein Release Kinetics and Initial Burst Release

**Figure 9:**
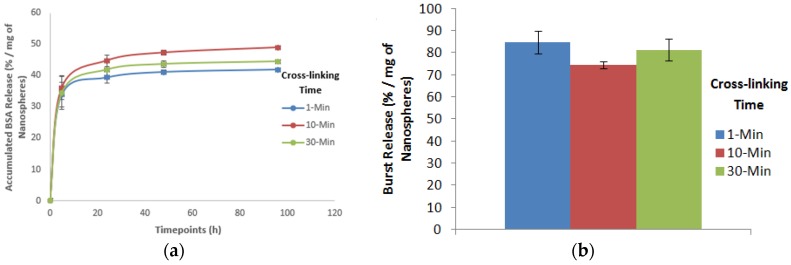
Effect of cross-linking time on the (**a**) protein release kinetics and (**b**) initial burst release of alginate nanospheres.

The effect of cross-linking time on the in vitro protein release kinetics and initial burst release is shown in Figure 9. The time of cross-linking varied from 1 to 30 min, while keeping the levels of other parameters constant, i.e., the alginate concentration and drying time at 5% (w/v) and 0 h respectively. The change in the cross-linking time appeared to have a very little effect on the protein release profile (Figure 9a). Specifically, at the initial timepoint (i.e., 5 h), increasing the cross-linking time from 1 to 10 min increased the protein release slightly and then decreased again when the cross-linking time increased to 30 min. The total protein release for different cross-linking times also showed similar patterns with the 10 min cross-linking time showing the highest total protein release compared to the other two cross-linking times. When the effect of the cross-linking time on the initial burst release was evaluated using Equation (1), it was found that increasing the cross-linking time decreased the initial burst release slightly (Figure 9b). Compared to the 1-min cross-linking time, both the 10 and 30-min cross-linking times showed less burst release with the 10-min cross-linking time showing the least.

#### 3.2.3. Encapsulation Efficiency

When the effect of the cross-linking time on the EE% was tested, increasing the cross-linking time showed no significant difference. With the increase of the cross-linking time from 1 to 10 min, the EE% increased and then decreased again for the 30-min cross-linking time. The EE% for 1, 10 and 30-min cross-linking times were 43 ± 7.50, 51 ± 8.09 and 43 ± 6.96 respectively.

### 3.3. Effect of Drying Time

#### 3.3.1. Morphology and Size of Nanospheres

**Figure 10:**
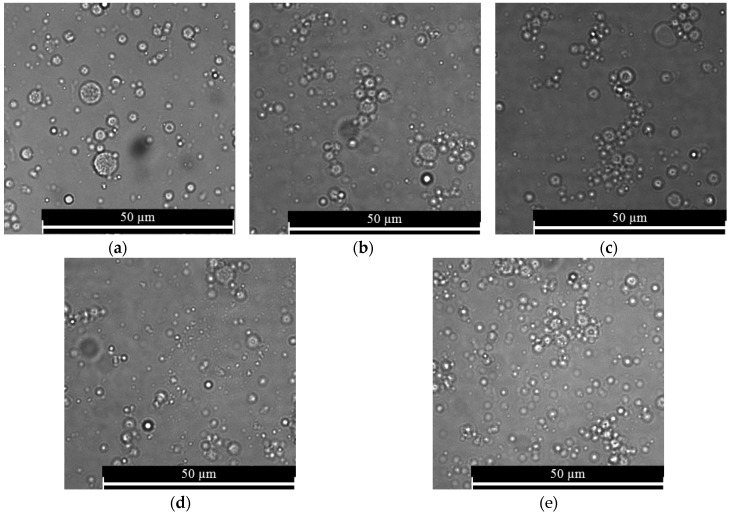
Morphology of alginate nanospheres prepared with (**a**) 0 h drying time; (**b**) 0.5 h drying time; (**c**) 1.5 h drying time; (**d**) 4.5 h drying time and (**e**) 24 h drying time. Optical microscope with a 63× magnification was used to take all of the micrographs.

**Figure 11:**
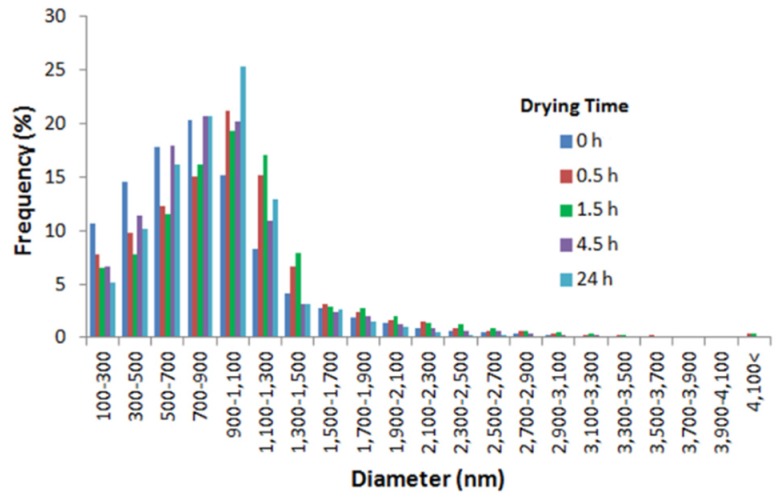
Size distribution pattern of alginate nanospheres prepared using different drying times.

**Figure 12:**
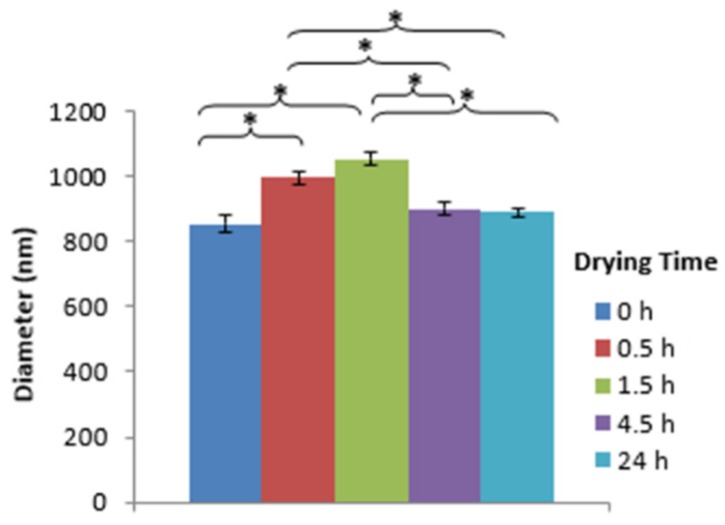
Comparison of the mean diameter of alginate nanospheres prepared using different drying times (*: Indicates significant difference among groups).

In order to determine the effect of drying time on the morphology, size, distribution pattern of nanospheres, the nanospheres were used either immediately after fabrication or after drying at 37 °C for several hours. During determination of the effect of drying time, the alginate concentration and cross-linking time were kept constant at 5% (w/v) and 10 min respectively. The nanospheres prepared using the 0-h drying time were discrete and spherical in shape, with nanospheres aggregation at some of the places (Figure 10a). However, with the increase of drying time, the tendency of nanospheres aggregation increased (Figure 10b–e). All of the drying time groups showed a normal size distribution pattern, implying the homogeneity of nanospheres (Figure 11). For the 0, 4.5 and 24-h drying times, most of the nanospheres were in the range from 700 to 900 nm. On the other hand, for the 0.5 and 1.5-h drying times, most of the nanospheres were in the range from 900 to 1100nm. With the increase of the drying time from 0 to 1.5 h, the size of nanospheres increased and then decreased again with the further increase of drying time (Figure 12). Compared to the 0-h drying time, both the 0.5 and 1.5-h drying time groups showed a significant increase (p ≤ 0.05) in the mean diameter. However, compared to the 0-h drying time, the effects of other drying times on the mean diameter of nanospheres were not significant. Furthermore, the 0.5 and 1.5-h drying times showed a significant increase in the mean diameter compared to the nanospheres prepared using the 4.5 and 24-h drying times.

#### 3.3.2. Protein Release Kinetics and Initial Burst Release

**Figure 13:**
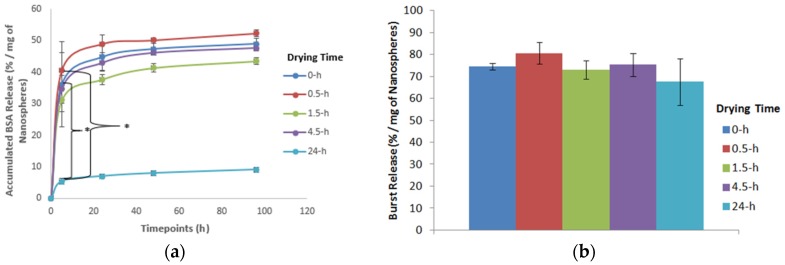
Effect of drying time on the (**a**) protein release kinetics and (**b**) initial burst release of alginate nanospheres (*: Indicates significant difference between groups).

The effect of drying time on the in vitro protein release kinetics and initial burst release is shown in Figure 13. The nanospheres were used either immediately after fabrication (without drying) or dried for different time periods (0.5, 1.5, 4.5 and 24 h). During the evaluation of the drying time effect, the levels of other parameters e.g., the alginate concentration and cross-linking time, were kept constant at 5% (w/v) and 10 min respectively. Increasing the drying time showed a significant difference in the protein release profile at the initial timepoint (Figure 13a). It was observed that among various drying time groups, drying of nanospheres for 24-h resulted in an incomplete protein release. This might be the reason for the 24-h drying time showing a significant decrease in the protein release compared to the 0 and 0.5-h drying times, at the initial timepoint (i.e., 5 h). For different drying time groups, the difference in the protein release after 5 h remained unchanged. 

When the effect of different drying times on the initial burst release was calculated (using Equation (1)), it was found that increasing the drying time showed no definite pattern on the initial burst release (Figure 13b). With the increase of drying time, only the 1.5 and 24-h drying time groups showed slightly less initial burst release compared to the undried nanospheres (0-h drying time group). On the other hand, the 0.5 and 4.5-h drying time groups showed more initial burst release compared to the undried nanospheres.

#### 3.3.3. Total Release

The drying time had a significant effect on the percentage of the total protein released at 96 h. The total protein release percentage was calculated based on how much BSA was released from all of the recovered nanospheres compared to the total amount of BSA added in each formulation. Drying of nanospheres for 24 h showed a significant decrease in the total protein release compared to all other drying time groups. The reason for the 24-h drying time group showing a significant decrease in the total protein release might be due to an incomplete protein release using this drying time.

## 4. Discussion

Alginate micro/nanospheres have been used as a protein delivery device in previous studies [11,12,13,14,15]. However, one of the major limitations of using alginate as a delivery device is the high initial burst release of the encapsulated proteins from alginate spheres within a very short time [15]. Based on previous studies, the release rate of the encapsulating molecules can probably be controlled by varying various process parameters, such as the polymer concentration [21,27,28,33], crosslinking time [29,30,31], drying time [32] of the micro/nanospheres. Here, this study examined whether it is possible to reduce the initial burst release of protein from alginate nanospheres by varying the alginate concentration, cross-linking time or drying time. The research hypothesis was, increasing the alginate concentration, cross-linking time or drying time allows for reduction of the initial burst release. To test the research hypothesis, the effect of alginate concentration, cross-linking time and drying time on the initial burst release was tested in this study.

### 4.1. Effect of Alginate Concentration

The concentration and MW of polymer have a considerable effect on the initial burst release and total release [23,34]. In this study, low-viscosity alginate was selected as the solution prepared from high viscosity alginate can become very viscous, and may cause damage to proteins from the high shear force generated during mixing [23]. During the investigation of the concentration effect, only the 3% and 5% low viscosity alginate were tested given that the alginate solution with a concentration higher than 5% became extremely viscous, which may cause the protein damage during mixing, as noted above.

Compared to the nanospheres prepared using the 3% alginate, the higher apparent contrast in the case of the 5% alginate might be due to the denser internal structure of alginate nanospheres at a higher alginate concentration. Furthermore, the increased alginate concentration showed a significant increase in the mean diameter of the nanospheres (Figure 4a), which is in agreement with the previous studies [28,35]. The studies found similar results explaining the phenomenon might be due to an increase in the solution viscosity at a higher alginate concentration resulting in larger aqueous droplets in emulsion.

In this study, when the in vitro protein release kinetics was determined (Figure 5a), increasing the alginate concentration showed no significant effect on the protein release at any of the timepoints. To evaluate if the change in the nanospheres’ size by varying the alginate concentration was affecting the release profile, the protein release kinetics for different alginate concentrations was normalized based on the relative size of nanospheres (see Supplemental Figure S4). However, for the same relative size of nanospheres increasing the alginate concentration to 5% also showed no significant effect in protein release at any of the timepoints. This finding implies that the change in the alginate concentration and nanosphere size (by varying the alginate concentration) have no significant effect on the protein release kinetics.

During evaluating the effect of alginate concentration on the initial burst release, it was found that increasing the alginate concentration from 3% to 5% significantly decreased the initial burst release (Figure 5b), which aligns with our research hypothesis. To determine if the change in the nanospheres’ size by varying the alginate concentration affects the release, the initial burst release was normalized again based on the nanospheres’ size to get the non-size effect of alginate concentration on the initial burst release (see Supplemental Figure S5). For the same relative size of nanospheres, increasing the alginate concentration to 5% showed a significant decrease in the initial burst release compared to the 3% alginate. Since increasing the alginate concentration showed a significant decrease in the initial burst release before (Figure 5b) and after normalization based on the relative size of nanospheres (Supplemental Figure S5), this finding confirmed that the change in nanospheres’ size had no significant effect on the initial burst release.

### 4.2. Effect of Cross-Linking Time

The swelling of micro/nanospheres also controls the release rate when a cross-linked hydrogel network is used as a delivery device [37]. Several studies have confirmed that increasing the ionic cross-linking time reduces the release rate of encapsulating molecules by increasing the cross-linking density and decreasing the micro/nanosphere swelling during incubation [29,30,31,38]. Since the effect of the ionic cross-linking time on the release rate of proteins from alginate micro/nanosphere was not investigated before, therefore, the effect of the cross-linking time on the initial burst release and protein release kinetics of alginate nanospheres was tested in this study. In order to test the effect of cross-linking time, the time of cross-linking varied from 1 to 30 min. Due to practical reasons, the 1-min cross-linking time was selected as the lowest value, as processing steps after adding the cross-linker (such as, hardening of nanospheres using isopropyl alcohol and transferring the nanospheres from the 24-well plate to Eppendorf tubes) requires at least 1-min. The intention of using the 1-min cross-linking time was to show how an incomplete cross-linking might affect the protein release from alginate nanospheres. The cross-linking time higher than 30 min was not tested in this study, because based on the literature, 30 min is the maximum time required to uptake Ca^2+^ by an alginate gel matrix [39].

With the increase of cross-linking time from 1 to 10 min, the mean diameter of the nanospheres decreased slightly and then increased again for the 30-min cross-linking time (Figure 8). This comparatively high mean diameter using the 30-min cross-linking time might be due to more cross-linking of the G-units of SA at a higher cross-linking time. However, increasing the cross-linking time did not have any significant effect on the mean diameter of nanospheres.

When the effect of the cross-linking time on the in vitro protein release kinetics was evaluated, the effect was not significant (Figure 9a). To determine the effect of the nanospheres’ size, when the protein release kinetics was normalized based on the relative size of nanospheres (see Supplemental Figure S6), like before (Figure 9a) increasing the cross-linking time for the same relative size of nanospheres also showed no significant effect on the protein release kinetics. These findings imply that the change in the ionic cross-linking time and the nanospheres’ size (by varying the cross-linking time) had no significant effect on the protein release kinetics. The degree of cross-linking by increasing the ionic crosslinking time was not probably enough to affect the release kinetics.

When the effect of the cross-linking time on the initial burst release was evaluated, it was found that increasing the cross-linking time slightly reduced the initial burst release (Figure 9b). This might be because with increasing the cross-linking time, the porosity of nanospheres slightly decreased due to a comparatively higher degree of cross-linking resulting in a less burst release. However, the effect of the cross-linking time on the initial burst release was not significant, which does not align with our research hypothesis. When the initial burst release for different cross-linking times was normalized to determine the non-size effect of the cross-linking time (see Supplemental Figure S7), increasing the cross-linking time for the same relative size of nanospheres also had no significant effect on the initial burst release. Increasing the cross-linking time showed no significant difference on the initial burst release before normalization based on the relative size of nanospheres as well (Figure 9b). Therefore, these findings imply that increasing the cross-linking time and the change in the nanospheres’ size by varying the cross-linking time had no significant effect on the burst release.

### 4.3. Effect of Drying Time

By increasing the alginate concentration and cross-linking time, it was possible to reduce the initial burst release to some extent. However, the initial burst release was still quite high (74.50 ± 1.53%). Based on a previous study, the degree of drying can influence the matrix porosity [32]. Earlier studies confirmed that while complete dehydration increases the burst release, due to the destruction of the structural integrity of microspheres, partial drying might reduce the release of the encapsulated molecules due to a decreased matrix porosity [32]. However, the effect of the drying time on the protein release from alginate nanospheres has not been investigated yet. Therefore, an effort was made in this study to determine the optimal drying time necessary to reduce the initial burst release. In order to better visualize the effect of the drying time on the protein release kinetics and initial burst release, drying times were selected based on the log interval as it allowed minimization of the number of groups necessary to determine the effect of a certain parameter on a certain outcome. Based on a previous study, freeze dried microspheres behave similar to wet microspheres and increase the protein release [40]. Therefore, instead of freeze drying, nanospheres were dried at 37 °C. Another reason of choosing 37 °C was that it matches with the physiological temperature, hence, the probability of protein denaturation should be minimal at this temperature.

In this study, compared to the undried nanospheres, the mean diameter of nanospheres prepared using the various drying times was comparatively high with a significant increase for the 0.5 and 1.5-h drying times (Figure 12). Based on literature, due to water loss during drying, the mean diameter of dried nanospheres should be less compared to their undried counterparts [28]. However, in this study, the 0.5 and 1.5-h drying time groups showed a significant increase in the mean diameter of nanospheres compared to the undried nanospheres. The nanospheres’ aggregation is probably the reason for getting significantly high mean diameters for the 0.5 and 1.5-h drying time groups compared to the 0-h drying time group. It may be when the external surface of nanospheres dried due to water loss, the distance between nanospheres decreased. This, in turn, might have formed new hydrogen bonds between alginate nanospheres due to the presence of hydroxyl groups in alginate, resulting in a comparatively high mean diameter of nanospheres. With a farther increase in the drying time, more water loss occurred from the internal surface of these initially aggregated nanospheres resulting in a farther decrease of the mean diameter.

When the in vitro protein release kinetics for different drying time groups was determined, increasing the drying time from 0 to 4.5 h showed no significant difference in the protein release at any timepoints (Figure 13a). Based on the literature, drying of microspheres reduces the water content from the gel network resulting in an increase in the gel concentration which eventually reduces the average pore size of the gel network [40,41]. Therefore, with the increase of drying time the protein release should decrease. However, no significant difference in the protein release was found for different drying time groups. The reason for not getting any significant difference in the protein release by varying the drying time might be because drying of nanospheres from 0 to 4.5 h was not enough to reduce the nanospheres’ porosity to such an extent that it affected the release rate. However, the effect of drying time on the nanospheres’ porosity was not tested in this study. In this study, the 24-h drying time was considered as a completely dehydrated group of nanospheres (as no change of nanospheres mass was observed after 24 h). Based on the literature, complete dehydration increases the release of encapsulating molecules due to the destruction of structural integrity [32]. This 24-h drying time was selected in order to confirm this negative effect of complete dehydration and to show why partial drying is better. Unexpectedly, the 24-h drying time group showed an incomplete protein release resulting in a significant decrease in the initial protein release, compared to the 0 and 0.5-h drying time groups. The reason of the 24-h drying time group showing incomplete protein release might be due to the nanospheres’ aggregation during drying, so the nanospheres did not disperse properly when placed in the release medium. Therefore, probably the release medium could not impregnate through all of the nanospheres preventing the BSA release. Another probable reason might be the shrinkage of the nanospheres to such an extent that it was irreversible when placed in the release medium. The protein release kinetics was normalized to determine if the change in the nanospheres’ size by varying the drying time affected the release kinetics. At the initial timepoint for the same relative size of nanospheres, not only the 0 and 0.5-h drying time groups, but also the 4.5-h drying time group showed a significant increase in the protein release compared to the 24-h drying time group (See Supplemental Figure S8). However, the 4.5-h drying time showed no significant difference in the protein release compared to the 24-h drying time before normalization based on the nanospheres’ size (Figure 13a). This finding implies that the change in the drying time might change the protein release kinetics significantly. However, the effect was obscured by the associated change in the nanospheres’ size.

To determine the effect of the drying time, when the initial burst release was calculated, it was found that increasing the drying time reduced the initial burst release slightly. However, the effect was not significant (Figure 13b), which is not in agreement with our proposed hypothesis. Since the nanospheres prepared using the 24-h drying time showed an incomplete protein release, therefore, the decrease in the initial burst release using the 24-h drying time was excluded from the final result. To determine the effect of the nanosphere’ size, the initial burst release for different drying time groups was normalized based on the relative size of nanospheres (See Supplemental Figure S9). Like before (Figure 13b), increasing the drying time for the same relative size of nanospheres showed no significant difference on the initial burst release (see Supplemental Figure S9). This result implies that the change in nanospheres’ size by varying the drying time had no significant effect on the initial burst release. 

While the results of this study confirm that it is possible to control the initial burst release by regulating various process parameters, there are some limitations in this study that beg for more studies in the future. For example, all the possible combinations of alginate concentration, cross-linking time and drying time were not tested in this study, as it becomes hard to predict the effect of a certain process parameter if other process parameters also vary concurrently. It might be interesting to see how other possible combinations of the alginate concentration, cross-linking and drying time, other than the ones tested in this study, affect the initial burst release. The timepoint selected to study the protein release studies were 5, 24, 48 and 96-h. The protein release at the 0-h time point was assumed to be zero. It would be interesting to see if any BSA was released in the supernatant immediately after fabrication (i.e., at the 0-h timepoint). In this study, during determination of the drying time, the 24-h drying time showed an incomplete protein release. More studies are needed to determine the optimal drying time necessary to reduce the initial burst release. It might be interesting to see what effect of increasing the drying time more than 4.5 h and less than 24 h might have on the initial burst release. In this study, it was not evaluated whether the process conditions (e.g., homogenization speed, centrifugation speed, and process parameters) affected the structure of the alginate and BSA. One of the approaches of determining the structure of the alginate and BSA might be using the NMR spectroscopy.

## 5. Conclusions

In this study, alginate nanospheres were developed as a protein delivery device by using a combination of emulsification and external gelation method. The aim of this study was to investigate the influence of alginate concentration, cross-linking time or drying time on the initial burst release of alginate nanospheres, thus allowing for the rigorous selection these parameters to reduce the initial burst release. The hypothesis was, increasing the alginate concentration, cross-linking time or drying time allows for reduction of the initial burst release. By increasing the alginate concentration, cross-linking time and drying time it was possible to reduce the initial burst release from 86 ± 3.62 to 73 ± 4.17%. Among the various process parameters, increasing the alginate concentration showed a notable effect on the initial burst release. The change of the size of nanospheres by varying the process parameters (e.g., alginate concentration, cross-linking time or drying time) showed no significant effect on the initial burst release.

## Figures and Tables

**Table 1:** Experimental design to determine the effect of various process parameters on the performance of bovine serum albumin (BSA) loaded alginate nanospheres.

Factors Tested	Alginate Concentration (%)	Cross-Linking Time (min)	Drying Time (h)
1st Step: Concentration Effect	3	10	0
	5	10	0
2nd Step: Cross-linking Time Effect	5	1	0
	5	10	0
	5	30	0
3rd Step: Drying Time Effect	5	10	0
	5	10	0.5
	5	10	1.5
	5	10	4.5
	5	10	24

## Supplementary Materials

The following are available online at https://www.mdpi.com/2079-4983/10/3/42/s1, Figure S1: Alginate interference during the Bradford assay, Figure S2: Effect of BSA on the size of alginate nanospheres, Figure S3: BSA release at different timepoints, Figure S4: Effect of alginate concentration on protein release kinetics normalized based on the relative size of nanospheres, Figure S5: Effect of alginate concentration on the initial burst release normalized based on the relative size of nanospheres, Figure S6: Effect of cross-linking time on protein release kinetics normalized based on the relative size of nanospheres, Figure S7: Effect of cross-linking time on the initial burst release normalized based on the relative size of nanospheres, Figure S8: Effect of drying time on protein release kinetics normalized based on the relative size of nanospheres, Figure S9: Effect of drying time on the initial burst release normalized based on the relative size of nanospheres.
