# Supplementary material for: Effect of Process Parameters on the Initial Burst Release of Protein-Loaded Alginate Nanospheres
Source: J Funct Biomater. 2019 Sep 16;10(3):42. doi: 10.3390/jfb10030042 (PMC6787618; doi:10.3390/jfb10030042)
Supplement: Supplementary file 1 [file jfb-10-00042-s001.pdf]

Journal of *Functional Biomaterials*

MDPI

Supplementary Materials

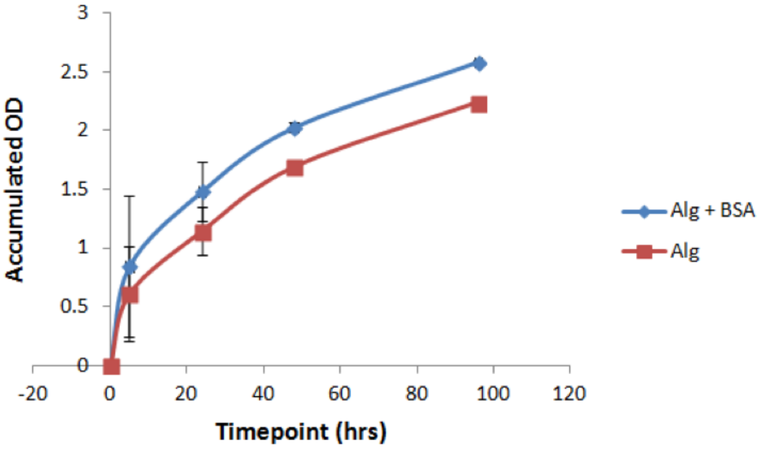

Figure S1. Alginate interference during the Bradford assay.

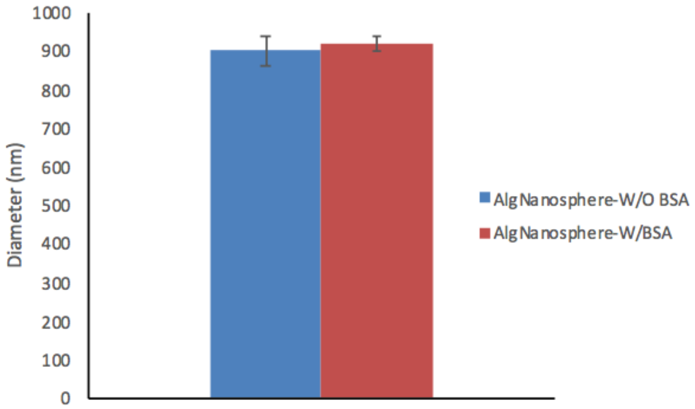

Figure S2. Effect of BSA on the size of alginate nanospheres.

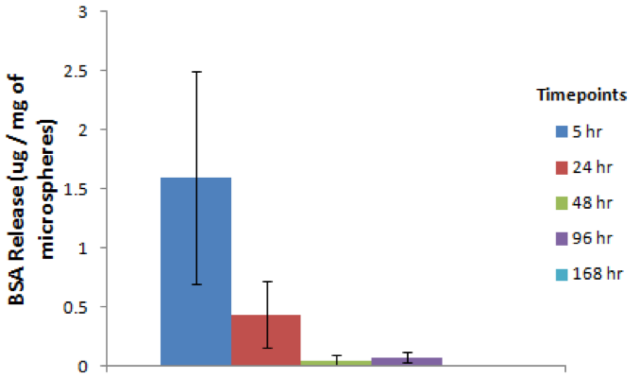

Figure S3. BSA release at different timepoints.

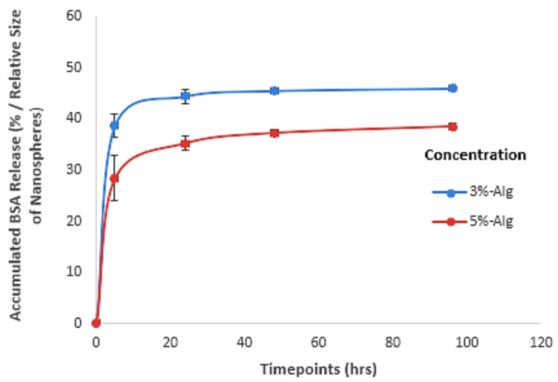

Figure S4. Effect of alginate concentration on protein release kinetics normalized based on the relative size of nanospheres.

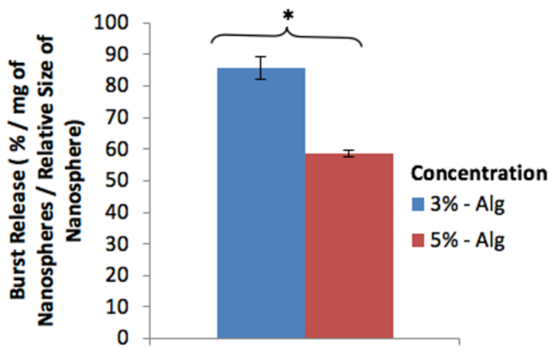

Figure S5. Effect of alginate concentration on the initial burst release normalized based on the relative size of nanospheres.

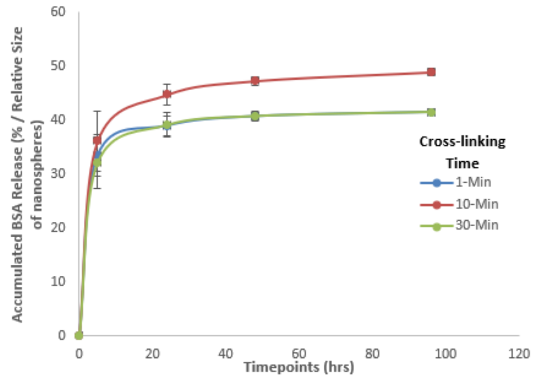

Figure S6. Effect of cross-linking time on protein release kinetics normalized based on the relative size of nanospheres.

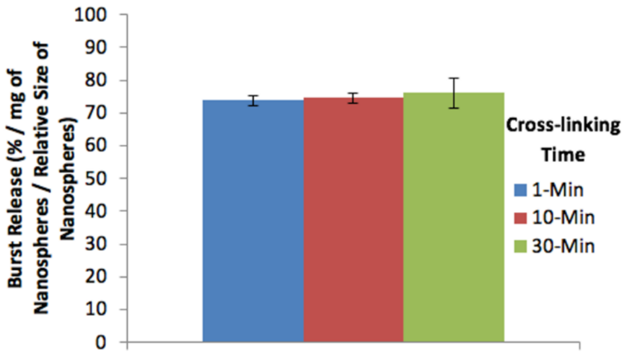

Figure S7. Effect of cross-linking time on the initial burst release normalized based on the relative size of nanospheres.

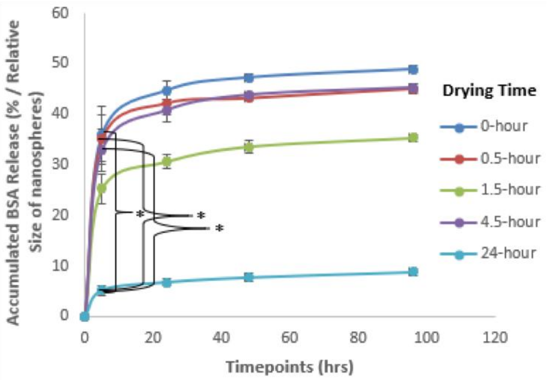

Figure S8. Effect of drying time on protein release kinetics normalized based on the relative size of nanospheres.

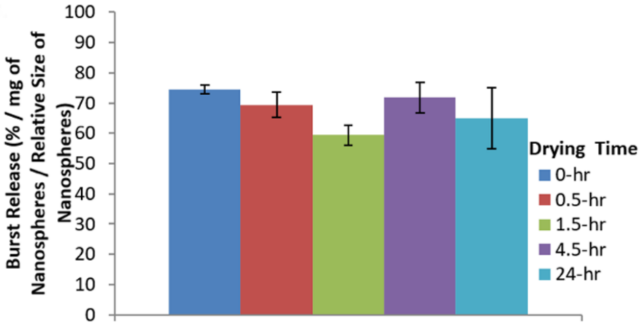

Figure S9. Effect of drying time on the initial burst release normalized based on the relative size of nanospheres.
